# Tackle Epithelial-Mesenchymal Transition With Epigenetic Drugs in Cancer

**DOI:** 10.3389/fphar.2020.596239

**Published:** 2020-11-27

**Authors:** Bo Dong, Zhaoping Qiu, Yadi Wu

**Affiliations:** ^1^ Department of Pharmacology and Nutritional Sciences, University of Kentucky School of Medicine, Lexington, KY, United States; ^2^ Markey Cancer Center, University of Kentucky School of Medicine, Lexington, KY, United States

**Keywords:** epigenetic modification, epithelial-mesenchymal transition, cell migration, metastasis, inhibitor

## Abstract

Epithelial-mesenchymal Transition (EMT) is a de-differentiation process in which epithelial cells lose their epithelial properties to acquire mesenchymal features. EMT is essential for embryogenesis and wound healing but is aberrantly activated in pathological conditions like fibrosis and cancer. Tumor-associated EMT contributes to cancer cell initiation, invasion, metastasis, drug resistance and recurrence. This dynamic and reversible event is governed by EMT-transcription factors (EMT-TFs) with epigenetic complexes. In this review, we discuss recent advances regarding the mechanisms that modulate EMT in the context of epigenetic regulation, with emphasis on epigenetic drugs, such as DNA demethylating reagents, inhibitors of histone modifiers and non-coding RNA medication. Therapeutic contributions that improve epigenetic regulation of EMT will translate the clinical manifestation as treating cancer progression more efficiently.

## Introduction

Epithelial-mesenchymal transition (EMT) is a phenomenon which involves the capacity of cells to interconvert between cellular states of the epithelial-mesenchymal axis and varying biological requirements (Bhatia et al., 2017; Yang et al., 2020). During this process, epithelial cells gradually dissolve cell-cell junctions, rebuild cell-matrix connections and attain mesenchymal phenotypes such as increased motility and invasiveness (Yuan et al., 2019). The resulting cells shift back, lose acquired mesenchymal traits and regain epithelial characteristics via mesenchymal-epithelial transition (MET) (Pei et al., 2019). Intriguingly, this cellular plasticity is very flexible, and transition *in vivo* appears to be partially performed; epithelial cells often undergo partial remodeling and display mixed combination of epithelial and mesenchymal features during EMT (Pastushenko and Blanpain, 2019). Such cellular conversions and the resultant heterogeneity are believed to equip cells with pliability to cope with different developmental and pathological occasions (Yang et al., 2020).

EMT can be activated by a cohort of soluble growth factors, extracellular matrix and micro-environmental conditions (Dongre and Weinberg, 2019). Outside mediators trigger within epithelial cells a large number of intracellular cascades, which ultimately refashion expressions of several EMT-TFs such as Snail/Slug, ZEB1/2 and Twist1/2 (Puisieux et al., 2014). These EMT-TFs cooperate in varied combinations to initiate EMT programs, orchestrating molecular interactions with regulatory elements at multiply layers (Lin et al., 2014). EMT programs include downregulation of genes associated with epithelial maintenance and upregulation of genes required for mesenchymal characteristics, doing so to confer cellular shift among the epithelial-mesenchymal spectrum (Pastushenko and Blanpain, 2019). For instance, a notable molecular hallmark of EMT is repression of E-cadherin, a transmembrane glycoprotein encoded by the epithelial marker gene CDH1 and essential for epithelial cell-cell junctions. Repression of E-cadherin, occasionally caused by genetic degeneration, can often be achieved through EMT-TFs binding to the E-cadherin promoter, which then recruit repressive transcriptional complexes.

Aside from being widely observed during development, wounding healing and tissue fibrosis, EMT is frequently associated with cancer progression (Brabletz et al., 2018). When carcinoma cells progress toward states of high-grade malignancy, EMT bestows cancerous cells with enhanced invasiveness that favors their spread through adjacent tissues and dissemination to distant sites, followed by recovery of proliferation potential as metastatic growth (Kröger et al., 2019). Furthermore, cancerous cells actively employing EMT often display elevated resistance to antitumor drugs and immunotherapy, and show properties reminiscent of cancer stem cell, a minority of phenotypically distinct cancer cell within a tumor mass capable of seeding new tumors (Shibue and Weinberg, 2017; Wilson et al., 2020). As a result, therapies that target subpopulations of cancerous cells undergoing EMT may generate consequential anticancer efficacy (Marcucci et al., 2016). Nonetheless, the exact role of EMT during cancer progression is far from clear (Nieto et al., 2016; Stemmler et al., 2019; Williams et al., 2019).

As our characterization of the EMT pathway expands, it becomes clear that activation and execution of EMT occurs as a result of genetic and epigenetic process (Skrypek et al., 2017). The word “epigenetic” refers to a layer of information existing beyond genetic data encoded in the genomic DNA sequence (Greally, 2018). Genomic DNA in eukaryotic cells interacts with histone proteins and RNA to form the chromatin, which serves as the crucial platform for epigenetic regulation of gene expression. Epigenetic regulation largely depends on alteration of chromatin states through regulators responsible for DNA methylation, post-translational modifications (PTMs) of nucleosomal histone tails and/or non-coding RNA modulation (Wen et al., 2009). Deregulation of epigenetic modifications constantly contribute to cellular disorders that lead to malignant transformation. A variety of epigenetic regulators are considered as critical requirements that interpret EMT signals passed from stimulators to transcription factors (Tam and Weinberg, 2013). Here, we outline the modulatory mechanisms of EMT during cancer progression, focusing on epigenetic regulation and compounds that target these epigenetic modifiers. An expanding description of the epigenetic regulations that underlie the contribution of EMT to cancer progression will provide momentous insights for “epigendrug” to treat cancer metastasis and therapeutic resistance.

## Epithelial-Mesenchymal Transition and DNA Methylation

DNA methylation involves a covalent attachment of a methyl group to the cytosine residues at the CpG-rich dinucleotide sequence (CpG island) through DNA methyltransferases (DNMTs). It is a stable and reversible process, with demethylation catalyzed by members of the ten-eleven translocation methyl cytosine dioxygenase (TET) (Rasmussen and Helin, 2016). DNA methylation usually modulates gene expression through the combination of two mechanisms. The presence of methyl groups within the CpG island can directly impair the recognition of transcription factors to CG-rich motifs, thus hinder gene expression. DNA methylation also serves as epigenetic marks to attract methyl-CpG binding-domain proteins (MBDs). MBDs interact with transcription factors, which recruit chromatin remodelers and histone-modifying proteins, thereby turning DNA methylation into activation or repressive signal depending on the cellular context. Upon induction of EMT, hypermethylation of the *CDH1* promoter through DNMTs, which are recruited by EMT-TFs, is constantly observed in a wide variety of cancer cells. In addition, the expression of EMT-TFs and other components of EMT regulatory elements are also regulated by DNA methylation. DNA demethylation promoted by TET is essential for MET in somatic cell reprogramming (Hu et al., 2014). The Mbd3/NuRD complex cooperates with TET2 and keeps cancer cells in a highly metastatic mesenchymal state. Combinatorial interference with their functions leads to MET and efficient repression of metastasis (Nihan Kilinc et al., 2020).

Decitabine, along with azacytidine, are two DNMT inhibitors that have displayed promising responses at sub-cytotoxic doses in myelodysplastic syndrome and are now FDA approved for use in this disease. Numerous studies indicated that treatment with decitabine leads to promoter demethylation and reactivates E-cadherin expression in certain cancer cell lines (Graff et al., 1995; Yoshiura et al., 1995; Nakayama et al., 2001; Maruya et al., 2004; Tsutsumida et al., 2004; Ishikawa et al., 2017). Conversely, other reports revealed conflicting results in which inhibition of DNMTs by decitabine facilitates the metastasis of cancer cells, concomitant with induction of pro-invasive genes (Ateeq et al., 2008; Lee et al., 2016). The first-generation of DNMT inhibitors, including decitabine, are all nucleoside analogs which incorporate into replicating DNA; they irreversibly attach with all DNMTs and induce their degradation. Therefore, treatment with these inhibitors generally leads to global loss of DNA methylation and displays broad cellular effects and limited clinical efficacy. Compared with these drugs, guadecitabine (SGI-110) is a prodrug of decitabine with improved pharmacologic and pharmacokinetic effects. It is under clinical investigation, alone or in combination with other therapies, in a number of solid and hematopoietic malignancies (Issa et al., 2015; Kantarjian et al., 2017). Studies show that dynamic changes in the DNA methylome occur in TGF-β-induced EMT. TGF-β can induce both expression and activity of DNMT1 and DNMT3A/B. Treatment with SGI-110 prevents TGF-β-induced EMT in ovarian cancer cells (Cardenas et al., 2014). Moreover, a reversible, non-DNA-incorporating DNMT1 inhibitor GSK3482364 was recently developed with significantly improved pharmacological properties (Gilmartin et al., 2020). This new class of DNA inhibitors is orally bioavailable and displays robust loss of DNA demethylation without the limitation of off-target effects. Preclinical and clinical characterization will be needed to determine whether these new DNMT1 inhibitors offer advantages over the classical DNA hypomethylating drugs. On the other hand, because DNMT inhibitors can reverse the hypermethylated state at the promoters of epithelial markers such as E-cadherin, they also induce the activation of pro-metastatic genes. For instance, a most recent work indicates that DNMT1 can be recruited by a subunit of the chromatin remodeling complex to the Snail promoter and suppress its transcription, therefore down-regulation of DNMT1 could also induce expression of EMT-TFs (Jiang et al., 2020). Consequently, targeting the DNA methylation readers like MBDs may provide a suitable alternative for the next generation of epigenetic therapies, and several studies have shown promising effects in terms of developing anticancer therapeutic strategies against the MBDs (Mahmood and Rabbani, 2019).

## Epithelial-Mesenchymal Transition and Histone Modification

### Acetylation

Lysine acetylation of histone tails, one of the most frequently found PTMs, is catalyzed by a variety of transcriptional co-activating complexes which contain lysine acetyltransferases (KATs). Because acetylation masks the positive charge on lysine residues, thereby weakening DNA-histone association and relaxing chromatin structure, histone acetylation is often associated with gene activation. The histone acetyltransferase adenovirus E1A-associated protein (p300) and CREB binding protein (CBP) are key transcriptional coactivator essential for a multitude of cellular processes and implicated in cancer progression. P300/CBP acetyltrasferases proximal region of the *Slug* promoter to enhance metastasis (Xin et al., 2019). In addition, p300/CBP-associated factor (PCAF)-dependent acetylation of the transcription factor intestine-specific homeobox (ISX) promotes its interaction with BRD4 and translocation of the resulting complex into the nucleus. This complex binds to promoters of EMT genes, where acetylation of histone three at lysine 9, 14, and 18 thus initiates transcriptional activation; these findings indicate that the p300/CBP-ISX-BRD4 axis mediates EMT and regulates tumor initiation and metastasis (Wang L.-T. et al., 2020).

Recently, several p300/CBP inhibitors have been developed, including natural products (Balasubramanyam et al., 2004), bi-substrate analogs (Lys-CoA) (Lau et al., 2000) and the widely utilized C646 (Bowers et al., 2010; Shrimp et al., 2016). C646 inhibits cell viability and promotes cell apoptosis in different cancer cells. I-CBP112, a specific and potent acetyl-lysine competitive protein-protein interaction inhibitor, targets the p300/CBP bromodomains (Picaud et al., 2015). I-CBP112 impairs aberrant self-renewal of leukemic cells and synergistically suppresses cell growth with the BET bromodomain inhibitor JQ as well as doxorubicin. Interestingly, NEO2734, a novel dual p300/CBP and BET bromodomain-selective inhibitor, inhibits growth and cancer progression (Yan et al., 2019; Morrison-Smith et al., 2020). However, most of these inhibitors lack potency or selectivity. A-485, a potent, selective and drug-like p300/CBP catalytic inhibitor, selectively inhibits proliferation across lineage-specific tumor types (Lasko et al., 2017).

### Deacetylation

Histone deacetylation reveals the positive charge of lysine and permits DNA-histone interaction. Therefore, histone deacetylation is believed to restrict gene transcription. Similar to KATs, histone deacetylases (HDACs) often form large complexes with other epigenetic modifiers to remove acetyl residue from their targets, serving as transcriptional repressors. KATs and HDACs together maintain histone acetylation at an appropriate intensity for proper gene regulation. Disturbing the balance between acetylation and deacetylation is highly associated with cancer progression. HDACs, in particular HDAC1 and HDAC2, are often recruited by EMT-TFs to gene promoter regions and form protein complexes to deacetylate histones and silence expression of epithelial gene factors. For instances, Snail mediates recruitment of the HDAC1/2 that contain Sin3A or NuRD repressor complexes to inhibit E-cadherin expression (Fujita et al., 2003; Peinado et al., 2004). Likewise, Twist1 and ZEB2 interact with the Mi2/NuRD complex and recruit them to the proximal regions of the E-cadherin promoter for transcriptional repression (Verstappen et al., 2008; Fu et al., 2011).

Recently, a number of HDAC inhibitors have been developed. They are divided into four basic structural classes including short chain fatty acids, hydroxamic acid–derived compounds, benzamides and cyclic peptides (Wawruszak et al., 2019). Among them, four drugs have been approved by the United States Food and Drug Administration, namely vorinostat, romidepsin, belinostat, and panobinostat. Vorinostat, romidepsin and belinostat are approved for the treatment of patients with cutaneous T-cell lymphoma (CTCL). Panobinostat is approved for the treatment of patients with multiple myeloma. However, HDAC inhibitors modulate EMT-related factors expression in cancer-type dependent manner. The effect of HDAC inhibitors such as Trichostatin A (TSA) and Valproic acid (VPA) on EMT seem controversial, with both pro-invasive and anti-invasive activities having been described. Some studies reveal that treatment with TSA reverses EMT via suppressing Slug in breast cancer, colorectal and prostate cancer cells (Wang et al., 2015; Wang X. et al., 2020). VPA inhibits glioma stem cells invasion through Snail and Twist1 downregulation and E-Cadherin re-localization (Riva et al., 2018), and blocks EMT in prostate carcinoma cells via repression and mono-ubiquitination of SMAD4 (Lan et al., 2016). Conversely, other reports demonstrated that TSA treatment with esophageal squamous cell carcinoma cells increases acetylation of RelA, thereby enhancing Slug expression which in turn induces EMT (Huang et al., 2020). In hepatocellular carcinoma cells, TSA/VA significantly induces the EMT phenotype with decreased E-cadherin, increases N-cadherin, vimentin, Snail and Twist1, and an enhanced capacity for cell migration and invasion (Yang et al., 2018). Similarly, HDAC inhibitors induce mesenchymal transition of the colon carcinoma cells, especially in the presence of TGF-β1 (Ji et al., 2015). They enhance invasion of human melanoma cells via upregulation of N-cadherin and inhibition of RhoA activity (Díaz-Núñez et al., 2016), and these inhibitors upregulate Snail through AKT/GSK-3β signals and post-transcriptional modification to promote EMT in colorectal cancer cells (Feng et al., 2015). Thus, the application of HDAC inhibitors for chemotherapeutic intervention requires careful caution. HDAC inhibitors represent the first successful anti-epigenetic therapy, so far, only four inhibitors have been approved by FDA. However, HDAC inhibitors emerge as a promising class of drug, particularly in combination with other agents.

### Acetylation Readers

By acting as epigenetic markers, histone acetylation also provides binding sites for acetyl reader proteins such as the bromodomain-containing proteins (BRDs). BRDs binds to ε-N-aminoacetyl groups of nucleosomal histone lysine and recruits histone modifiers and transcriptional/remodeling factors to gene promoters, thereby upregulating or repressing gene expression in response to different biological signals. Ever increasing studies in different cancer cells have demonstrated the contribution of BRDs to cancer progression, reinforcing the therapeutic value of acetyl reader proteins (Belkina and Denis, 2012).

Recently, we found that acetylation of Twist1 by Tip60 acetyltransferase recruits BRD4 to the promoter and enhancer of *WNT5A* to modulate its expression. Pharmacologic inhibition of the Twist1-BRD4 interaction with JQ1 or MS417 reduces WNT5A expression and suppresses cell invasion, CSC-like properties, and tumorigenicity in basal-like breast cancer cell lines (Shi et al., 2014). Similarly, the acetylated SPZ1-Twist1 complex recruits BRD4 to upregulate expression of vascular endothelial growth factor, thus enhancing RNA-Pol II-dependent transcription and inducing metastasis (Wang L.-T. et al., 2019). Moreover, inhibition of BRD4 decreases Snail expression by diminishing the protein kinase D1 mediated proteasome degradation pathway. BRD4 inhibition also suppresses the expression of Gli1 which is required for transcriptional activation of Snail, indicating that BRD4 controls malignancy of breast cancer cells via both transcriptional and post-translational regulation of Snail (Lu et al., 2020). In renal cell carcinomas, inhibition of BRD4 by genetic knockdown or JQ1 prevents cell proliferation and EMT, and induces NF-κB-NLRP3-Caspase 1-dependent pyroptosis, providing evidence for BRD4 being a potential target and JQ1 as a therapeutic agent for renal cell carcinomas (Tan et al., 2020). Interestingly, we discovered that JQ1 has different effects on cell migration in AR-positive and AR-negative prostate cancer cells, which indicates that the function of BRD4 is cellular-context dependent (Wang J. et al., 2020). Owing to the experimental tractability and high potentials to bind small molecules, a variety of BRD inhibitors have been discovered that display good anti-inflammatory and anticancer activities. Several BRD inhibitors including OTX015, BET-d246, ABBV-075 have since advanced to early clinical trials to determinate safety, tolerability, and efficacy as a monotherapy and in combination with other agents (Mohammad et al., 2019). A major problem for the clinical translation of BRD inhibitors has been the relative immature understanding of BRD cellular functions and their relevance in disease; increasing validation and optimizing clinical comprehension is therefore a promising scenario for advanced cancer therapy (Cochran et al., 2019).

### Methylation

Histone lysine methylation is catalyzed by lysine methyltransferases (KMTs), which use S-adenosylmethionine as the methyl donor to align methyl groups to lysine residues. Unlike acetylation, histone methylation keep the charge of the histone protein and lysine residues can be monomethylated, dimethylated, or trimethylated, respectively. Histone methylation directly recruit or inhibit the recruitment of histone-binding proteins. For instance, H3K9 and H3K27 methylation are usually associated with transcriptional activation, while H3K79 is often linked with gene repression. Among all these KMTs, development of inhibitors of EZH2 (Enhancer of zeste homolog 2) and DOT1L (Disruptor of telomeric silencing-1-like) have advanced to clinical trials. EZH2, the catalytic subunit of the Polycomb Repressive Complex 2 (PRC2), promotes gene silencing by catalyzing mono/di/tri-methylation of H3K27, a major transcription repressive modification. Many studies show that EZH2 mediates transcriptional silencing of E-cadherin by trimethylation of H3K27, which establishes a functional link between dysregulation of EZH2 and aberrant modulation of EMT programs (Cao et al., 2008; Herranz et al., 2008). Different types of EZH2 inhibitors have been developed, and a number of drugs are ongoing in clinical trials for different cancer types. DZNep, the first EZH2 inhibitor, globally inhibits histone methylation but is not specific to EZH2 (Miranda et al., 2009). Tazemetostat (Tazverik™) is a selective and orally bioavailable EZH2 inhibitor. In January 2020, it was approved by FDA for adults and pediatric patients aged 16 years and older with metastatic or locally advanced epithelioid sarcoma not eligible for complete resection (Duan et al., 2020). Recently, many new drugs targeting EZH2 are under development and evaluation in clinical trials including combinational treatment (Duan et al., 2020). Combining EZH2 inhibitors with other treatments such as immune therapy, conventional chemotherapy, and targeted therapy might have a synergic effect on tumor metastasis.

DOT1L catalyzes the methylation of an active transcription mark histone H3K79, which is crucial for tumor development (Okada et al., 2005). During reprogramming of embryonic stem cells, several EMT-TFs associated with DOT1L are modified by H3K79me (Onder et al., 2012). In breast cancers, DOT1L forms a transcriptionally active complex with c-Myc and p300 to facilitate H3K79 methylation and acetylation in the promoter regions of EMT-TFs and enhance their de-repression, consequently promoting EMT-induced CSC properties (Cho et al., 2015). EPZ004777, the first reported DOT1L inhibitor, inhibits self-renewal and metastatic potential of breast cancer (Wong et al., 2015). In addition, treatment with a highly selective inhibitor EPZ5676 or small interfering RNA against DOT1L reduces expression of Snail and Twist1, and inhibits TGF-β1 and serum-induced activation of renal interstitial fibroblasts and EMT (Liu et al., 2019). Targeting DOT1L by a novel psammaplin A analog augments expression of E-cadherin and suppresses N-cadherin, ZEB1, and vimentin expression and inhibits growth and metastasis of triple-negative breast cancers (Byun et al., 2019). These studies provide evidence that targeting DOT1L-mediated H3K79 methylation may be a promising strategy for the treatment of cancer.

Moreover, many other KMTs such as G9a have been characterized with respect of EMT. G9a is a major KMT responsible for the transcriptionally repressive modification of H3K9. G9a is preferentially expressed in aggressive lung cancer cells and its elevated expression correlates with poor prognosis. G9a represses a cell adhesion molecule EPCAM by catalyzing H3K9me2 on its promoter to stimulate EMT and cancer metastasis of lung cancer cells (Chen et al., 2010). In breast cancer cells, G9a is recruited to the *E-cadherin* promoter for transcription silencing by interaction with Snail. Inhibiting G9a reduces promoter H3K9me2 as well as DNA methylation and further abrogates EMT and tumor metastasis (Dong et al., 2012). A similar mechanism was also reported in head and neck squamous cell carcinoma (Liu et al., 2015). In recent years, many potent G9a inhibitors have been discovered based on their binding modes: 1) substrate competitive inhibitors, such as UN0638; 2) the SAM (S-adenosyl -methionine) cofactor competitive inhibitors; 3) mechanism remains unclear (Cao et al., 2019). BIX01294 was the first small molecular G9a inhibitor identified by high-through screening in 2007 (Kubicek et al., 2007). Treatment with BIX01294 not only inhibits cell proliferation but also affects breast cancer migration (Kim et al., 2018; Chae et al., 2019). The recently reported discovery of UNC0638, an inhibitor of G9a and GLP (a KMT which shares similar functional domains to and forms a heterodimer with G9a) has excellent potency and selectivity. UNC0638 treatment decreases global H3K9me2 levels and reduces H3K9me2 at promoters of G9a-regulated endogenous genes (Pan et al., 2015). In addition, UNC0638 activates E-cadherin expression and reverses EMT, thereby inhibiting tumor growth in pancreatic cancer cells (Vedadi et al., 2011). To date, there are no G9a inhibitors in clinical trials.

### Demethylation

Histone lysine-specific demethylase 1 (LSD1) functions as an epigenetic regulator by removing methyl groups from the transcription-activating H3K4 or the repressive H3K9 through an amine oxidase reaction (Shi et al., 2004; Metzger et al., 2005). Overexpression of LSD1 has been detected in a variety of tumors and is associated with metastasis and drug resistance, therefore LSD1 is regarded as a biomarker of poor prognosis. LSD1 often takes part in different chromatin-remodeling protein complexes and targets distinct proteins that regulate tumor progression. We found that the amine oxidase domain of LSD1 interacts with the SNAG domain of Snail (Lin et al., 2010). SNAG, similar to the histone H3 tail, acts as a molecular hook to recruit LSD1 and form the Snail1-LSD1-CoREST complex to repress E-cadherin expression and enhance cell migration (Lin et al., 2010). Another study indicated that Snail recruits LSD1 on epithelial gene promoters for H3K4 me2 demethylation, thereby silencing their expression and promoting EMT (Lin et al., 2010). By contrast, LSD1 can inhibit cellular invasiveness and exerts a metastasis inhibition function by acting as an integral component of the Mi-2/NuRD complex (Wang et al., 2009), or forming a complex with Sin3a to maintain the epithelial states (Yang et al., 2018). These contradictory findings indicate that LSD1 regulates EMT through diverse mechanisms possibly because of multiple histone lysine sites it can modify when combined with different complexes.

Specific modulation of LSD1 activity with inhibitors has been a promising therapeutic approach. Multiple LSD1 inhibitors have been discovered including irreversible and reversible inhibitors. For instance, treatment with Parnate, an enzymatic inhibitor of LSD1, or TAT-SNAG, a cell-permeable peptide corresponding to the SNAG domain of Slug, blocks the Snail/Slug-LSD1 interaction and suppresses the motility and invasiveness of cancer cells of different origins and genetic background (Ferrari-Amorotti et al., 2013). Pretreatment of cancer cells with the LSD1/2 inhibitor Tranylcypromine (TCP), an irreversible inhibitor, attenuates Snail-mediated of epithelial gene downregulation and upregulation of mesenchymal marker genes. Combined treatment with TCP and TSA completely abrogates EMT (Javaid et al., 2013). SP-2509 is a highly potent, reversible, and non-competitive LSD1 inhibitor which was discovered through high-throughput virtual screening (Sorna et al., 2013). Treatment of SP-2509 suppressed prostate cancer migration, and metastasis independent of AR expression (Wang et al., 2020). For now, several LSD1 inhibitors including TCP, ORY-1001, GSK2879552, IMG-7289, INCB059872, and ORY-2001(Vafidemstat) are being tested in clinical trials targeting anti-tumor traits, alone or in combined treatment for cancer therapy (Fang et al., 2019). Therefore, determination of their effects on EMT traits represents another potential validation of novel epigenetic drugs.

## Epithelial-Mesenchymal Transition and Non-Coding RNA: MicroRNA and Long Non-Coding RNA

Over recent years, more and more non-coding RNAs (ncRNAs) have been identified as crucial regulators for a variety of cellular functions including tumor progression and EMT (Anastasiadou et al., 2018). As a result, much attention has been paid on using ncRNAs as potential targets for tumor treatment including for EMT interference (Matsui and Corey, 2017). MicroRNAs (miRNAs) are small endogenous ncRNAs consisting of about 19–25 nucleotides that play important roles in gene expression, and underlie physiological and pathological processes (Bracken et al., 2016). MiRNAs can promote or suppress EMT by modulating the expression of EMT-TFs or its regulatory elements. Therefore, miRNA therapeutics could be achieved by either administration of miRNA mimics or anti-miRNAs. MiRNA mimics are synthetic double-stranded small RNA molecules that have the same sequences as the corresponding miRNAs and can restore their normal expression and function. By contrast, anti-miRNAs have complementary sequences so as to bind to target miRNAs and block their function.

### MicroRNA

miR-21 is an oncogenic miRNA with anti-apoptotic roles that is overexpressed in tumors compared with normal tissues, and a potent inducer of EMT (Pan et al., 2010). Recent reports revealed that miR-21 governs lung cancer cell progression and EMT through regulation of PTEN/Akt/GSK3β signaling cascade (Dai et al., 2019). Treatment with a specific inhibitor to miR-21, AC1MMYR2, blocks procession of pre- miR-21 to mature miR-21. This blockade causes EMT reversal and suppresses tumor growth and invasion, partly through upregulation of miR-21 targets as demonstrated in epithelial tumor cells and orthotopic nude mouse models (Shi et al., 2013). A selective peptide identified by phage display downregulates miR-21 expression through binding to pre-miR-21 which hinders Dicer processing. By antagonizing miR-21 function, this peptide is able to increase the expression of target proteins that increase apoptosis and suppress cell proliferation, invasion and migration (Bose et al., 2015). Moreover, treatment of sophocarpine, a naturally occurring tetracyclic quinolizidine alkaloid with promising therapeutic properties, blocks Dicer-mediated miR-21 maturation and leads to EMT reversal and growth inhibition in head and neck squamous cell carcinoma (Liu et al., 2017). Similarly, another natural product, butylcycloheptyl prodiginine also binds and inhibits Dicer-mediated processing of pre-miR-21 and selectively arrests growth of colon cancer cells (Matarlo et al., 2019).

Unlike aberrant upregulation of miR-21, expressions of miR-34 and miR-200 are repressed in a broad range of cancers. A dynamic model of the core EMT regulatory circuit was recently proposed based on two mutually inhibiting microRNA-TF loops, miR-34 (a,b,c)/Snail and miR-200/ZEB (Lu et al., 2013; Jia et al., 2019). Snail and ZEB repress the transcription of miR-34a/b/c and miR-200, respectively, while miR-34a/b/c and miR-200 bind with the corresponding mRNAs to inhibit protein levels of Snail and ZEB, by blocking their translation and/or enhancing their degradation (Park et al., 2008; Schliekelman et al., 2011; Siemens et al., 2011). Treatment with MRX34, a miR-34a mimic encapsulated in lipid nanoparticles, leads to significant tumor regression in the aggressive Kras:Trp53 NSCLC mouse model (Wiggins et al., 2010). Combination of miR-34a with another repressor miRNA let-7 using the same lipid particles, individually or in combination, results in synergistic potentiation of the anti-proliferative effects of erlotinib, the epidermal growth factor receptor inhibitor (Stahlhut and Slack, 2015). However, despite its promise, in several preclinical studies of different cancers, MRX34 clinical phase I trials have been halted because of immune-related serious adverse events. MiR-200 is another miRNA that has been targeted in preclinical studies. It was demonstrated that delivery of miR-200 mimics using DOPC lipid nanoparticles markedly reduces metastasis and angiogenesis and induces vascular normalization in several types of cancer (Pecot et al., 2013). In an orthotopic mouse model of lung cancer, treatment of tumors with miR-200c mimics enhances radiosensitivity, suggesting combination of miR-200 with radiation represents a therapeutic strategy in the future (Cortez et al., 2014). Although these studies have not examined directly whether therapeutic delivery of miR-200 results in EMT inhibition or reversal, a recent study revealed that nanoparticle delivery of miR-506, a miRNA that prevents TGFβ-induced EMT by targeting Slug, effectively suppresses tumor growth and blocks EMT in two ovarian cancer models (Yang et al., 2013). Future studies are needed for development of novel miRNA inhibitors and mimics with efficient delivery systems and to validate their clinical value relevant to EMT and tumor progression.

### LncRNA

Long ncRNAs (lncRNAs) belong to a class of ncRNA that are longer than 200 bp and have weak protein-coding potential. Many studies show that lncRNAs have significant roles in diverse biologic processes that affect gene expression at multiple levels, from gene transcription to protein expression; hundreds have been identified to be aberrantly expressed in human cancers (Anastasiadou et al., 2018; Slack and Chinnaiyan, 2019). In particular, an increasing number of lncRNAs are reported to be implicated in tumor progression and metastasis (Slack and Chinnaiyan, 2019). A major mechanism by which lncRNAs regulate EMT is by epigenetically silencing EMT-related genes such as E-cadherin via recruitment of transcriptional repressors like PRC2 (Lin et al., 2018). Alternatively, lncRNAs contribute to the modulation of EMT by acting as competing endogenous RNAs (ceRNAs) for EMT-associated miRNAs and impeding the interaction of these miRNAs with their target molecules in a stoichiometric manner (Wang L. et al., 2019; Fang et al., 2020). In terms of the canonical miR-200/ZEB double negative feedback loops which are critical to the induction of EMT, a number of lncRNA including lncRNA-ATB, H19 and HULC upregulate ZEB1/2 by competitively binding the miR-200 family and induce EMT (Yuan et al., 2014; Liang et al., 2015; Expósito-Villén et al., 2018; Liu et al., 2019; Ashrafizadeh et al., 2020). Likewise, transforming growth factor beta-induced (TGFBI) functions as a ceRNA for miR-21 and leads to de-repression of its endogenous targets FOXP1, a potent transcriptional inducer of EMT in A549 cells (Liu et al., 2019).

LncRNAs can be therapeutically targeted by a variety of approaches such as RNA interference, antisense oligonucleotides (ASO)-based therapy, lncRNA mimics and small molecule inhibitors. Several studies showed the success of LncRNA treatment in cancer therapy. For example, BC-819, a double stranded DNA plasmid that targets the expression of diphtheria-toxin gene under the control of H19 regulatory sequence, has been tested in clinical phase 2b trails in different cancers with promising therapeutic benefits (Hanna et al., 2012; Gofrit et al., 2014; Lavie et al., 2017). Metastasis associated lung adenocarcinoma transcript 1 (MALAT1) is extremely abundant in many human cell types and highly conserved across mammalian species (Sun and Ma, 2019). Subcutaneous injection of MALAT1-targeting ASO in a mouse model of metastatic luminal B breast cancer resulted in the formation of cystic and non-metastatic tumors (Arun et al., 2016). *In vivo* injection of MALAT1 ASOs also successfully decreased the expression of MALAT1 and suppressed lung cancer progression, which suggests that targeting MALAT1 in lung cancer patients is a potential and promising clinical therapy (Gutschner et al., 2013). Due to its high potency, RNA technology provides immense therapeutic promise. The development of new technologies will help to bring lncRNA-based therapies closer to the clinic (Khorkova and Wahlestedt, 2017).

## Discussions

**FIGURE 1:**
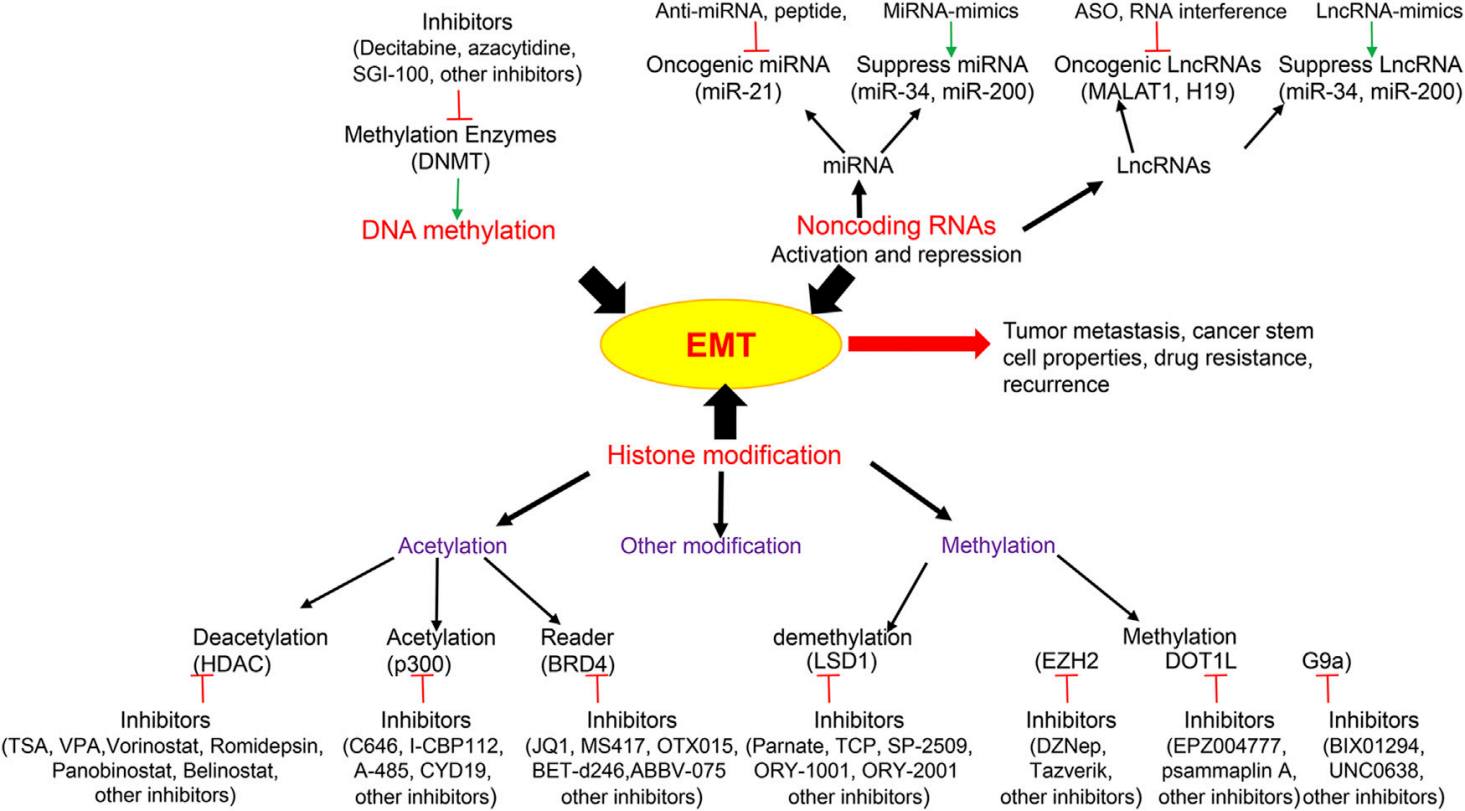
Epigenetic modifications and related key inhibitors involved in Epithelial-Mesenchymal Transition (EMT). Epigenetic regulations including DNA methylation, histone modification and non-coding RNAs are integral to EMT which contribute to tumor metastasis, cancer stem cell properties, drug resistance and recurrence. Epigenetic drugs are illustrated to inhibit EMT including histone deacetylase (HDAC) inhibitor, histone acetylase inhibitor, acetylation reader inhibitor, histone methyltransferase inhibitor, histone demethyltransferase inhibitor, DNA methyltransferase (DNMT) inhibitor, and non-coding RNA activation and repression. LncRNA, Long non-coding RNAs; MALAT1, Metastasis Associated Lung Adenocarcinoma Transcript 1; ASO, antisense oligonucleotides; BRD4, Bromodomain-containing protein 4; LSD1, Lysine-specific demethylase 1; EZH2, Enhancer of zeste homolog 2; DOT1L, DOT1 Like Histone Lysine Methyltransferase.

Over the past a few years, EMT research has experienced an explosive growth and emerged as a key event for evasion to various types of cancer therapy (Boumahdi and de Sauvage, 2020). Along with ever broadening comprehension of the mechanisms that control this phenotypic shift, its close connection to CSCs, drug resistance and other properties associated with tumor invasion and metastasis, there is rising interests to exploit EMT as a potential therapeutic target (Figure 1). The development of specific drugs against EMT is continuously increasing (Voon et al., 2017). Because of the reversibility of the epigenetic marks and the enzymatic nature of the regulators, EMT as a reversible and transient process, with intimate connection between EMT-TFs and chromatin-remodeling enzymes, targeting the epigenetic enzymes to reverse the EMT process is an efficient and promising approach. However, the clinical advancement for targeted anti-EMT therapy is still a field in its infancy (Brabletz et al., 2018), and there are only a few studies based on treatments that directly target EMT (Santamaria et al., 2017). Several problems need to be overcome to treat the EMT more efficiently and specifically by epigenetic inhibitors: First, the mechanisms responsible for the initiation, maintenance and alteration of the EMT schedule remain largely unknown and need further investigation; Second, the epigenetic regulators that contribute to the spectrum of EMT, particular during the intermediate states must be defined; Third, do the EMT-TF domains directly or indirectly interact with epigenetic enzymes; would disruption between EMT-TFs and epigenetic regulators reverse EMT-related properties? Fourth, epigenetic enzymes have variability based on the cellular context, that is, they can have an opposite function by formation distinct complexes in different cancers; therefore, how can we formulate EMT treatments with precision using an epigenetic inhibitor? Finally, how do EMT-TFs and the epigenetic network functionally cooperate with each other? How do they specifically intertwine to modify the EMT?

In all, additional knowledge of the EMT-TF epigenetic network will provide new efficient and specific avenues to enhance the clinical use of “epigendrug” to tackle EMT in cancer in the future.

## Author Contributions

All authors contributed to the manuscript content and editing for this review. YW provided supervision and financial support.

## Funding

Our research was supported by the Shared Resources of the University of Kentucky Markey Cancer Center (P30CA177558). Our research was also supported by grants from American Cancer Society Research Scholar Award (RSG13187) and NIH (P20GM121327 and CA230758) to YW.

## Conflict of Interest

The authors declare that the research was conducted in the absence of any commercial or financial relationships that could be construed as a potential conflict of interest.
